# PlexinD1 Is a Novel Transcriptional Target and Effector of Notch Signaling in Cancer Cells

**DOI:** 10.1371/journal.pone.0164660

**Published:** 2016-10-17

**Authors:** Michael Rehman, Sreeharsha Gurrapu, Gabriella Cagnoni, Lorena Capparuccia, Luca Tamagnone

**Affiliations:** 1 Cancer Cell Biology Laboratory, Candiolo Cancer Institute-FPO, IRCCS, Candiolo, Italy; 2 Department of Oncology, University of Torino, Torino, Italy; Southern Illinois University School of Medicine, UNITED STATES

## Abstract

The secreted semaphorin Sema3E controls cell migration and invasiveness in cancer cells. Sema3E-receptor, PlexinD1, is frequently upregulated in melanoma, breast, colon, ovarian and prostate cancers; however, the mechanisms underlying PlexinD1 upregulation and the downstream events elicited in tumor cells are still unclear. Here we show that the canonical RBPjk-dependent Notch signaling cascade controls PlexinD1 expression in primary endothelial and cancer cells. Transcriptional activation was studied by quantitative PCR and promoter activity reporter assays. We found that Notch ligands and constitutively activated intracellular forms of Notch receptors upregulated PlexinD1 expression; conversely RNAi-based knock-down, or pharmacological inhibition of Notch signaling by gamma-secretase inhibitors, downregulated PlexinD1 levels. Notably, both Notch1 and Notch3 expression positively correlates with PlexinD1 levels in prostate cancer, as well as in other tumor types. In prostate cancer cells, Sema3E-PlexinD1 axis was previously reported to regulate migration; however, implicated mechanisms were not elucidated. Here we show that in these cells PlexinD1 activity induces the expression of the transcription factor Slug, downregulates E-cadherin levels and enhances cell migration. Moreover, our mechanistic data identify PlexinD1 as a pivotal mediator of this signaling axis downstream of Notch in prostate cancer cells. In fact, on one hand, PlexinD1 is required to mediate cell migration and E-cadherin regulation elicited by Notch. On the other hand, PlexinD1 upregulation is sufficient to induce prostate cancer cell migration and metastatic potential in mice, leading to functional rescue in the absence of Notch. In sum, our work identifies PlexinD1 as a novel transcriptional target induced by Notch signaling, and reveals its role promoting prostate cancer cell migration and downregulating E-cadherin levels in Slug-dependent manner. Collectively, these findings suggest that Notch-PlexinD1 signaling axis may be targeted to impair prostate cancer cell invasiveness and metastasis.

## Background

Plexins are cell surface receptors for extracellular signals of the semaphorin family[1]. Mammalian semaphorin genes are divided into five classes—Class 3 to 7, which bind directly to Plexins or in association with co-receptor molecules. Semaphorin signaling has been implicated in a wide range of functions in development and in disease, ranging from axon guidance during morphogenesis to cancer progression [2]. Semaphorin signaling has been found to regulate multiple hallmarks of cancer such as invasion, angiogenesis, and proliferation among others [3]. Depending on the context of available signaling intermediates, they are found to play either a tumor suppressive or a pro-tumorigenic role. For instance, in endothelial cells Sema3E and its specific receptor PlexinD1 inhibit cell-substrate adhesion [4] and exert an anti-angiogenic function, while in cancer cells they have been shown to have a pro-tumorigenic role [5]. In particular, Sema3E and its mature isoform Sema3E-p61 regulate migration and invasion of melanoma, colon, lung, and ovarian cancer cells, and Sema3E-PlexinD1 signaling was reported to promote invasive/metastatic phenotype [5,6]. We reported previously that in certain cancer cells Sema3E-PlexinD1 can transactivate ErbB2 signaling, promoting the invasive/metastatic phenotype [5]. Other studies showed Sema3E-dependent activation and nuclear translocation of the transcription factor Snail in ovarian cancer cells [6], or rather dependence-receptor features of PlexinD1 in breast cancer cells [7].

Notably, PlexinD1 is expressed at low levels in adult tissues, but it is typically overexpressed in multiple types of human cancer [5,6,7,8,9], both in tumor cells and in tumor vasculature; yet, the mechanisms sustaining this expression have not been elucidated. PlexinD1 is also remarkably expressed in endothelial cells, where it is required for vascular patterning in angiogenesis [10]. Recently, it was reported that VEGF positively controls the expression of PlexinD1 in endothelial cells of actively sprouting blood vessels in retinal development [11]. Moreover, PlexinD1 expression was induced by hypoxia in post-ischemic regenerating vessels [12]. However, the relevance of this pathway in other tissues has not been investigated. Notably, in the vasculature of developing retina Notch signaling was associated in one study with reduced PlexinD1 expression [11], while in a different developmental system this was not confirmed [13]. There are four known Notch receptors (Notch1—Notch4); upon binding of ligands of the DSL family—Jag1, Jag2, Dll1, Dll3, Dll4—Notch receptors undergo subsequent cleavage by TACE and gamma secretase. Once released in the cytosol, Notch intracellular domain (N-ICD) translocates into the nucleus, where it forms complex with RBPjk/CBF1 transcription factor to regulate gene transcription. Notch family members are overexpressed in several human tumors, and their signaling cascade is often activated by an increased load of notch ligands in the tumor microenvironment, for example in response to inflammation or hypoxia [14,15].

Sema3E-PlexinD1 signaling was recently reported to be upregulated in prostate cancer and regulate cell migration [8]. Prostate cancer is one of the most frequently diagnosed cancers in men. While relatively tractable in its early stage, in the advanced stage this cancer has a poor prognosis due to regional invasion and metastatic dissemination. The implicated mechanisms are under intense investigation. Similar to other tumors, in prostate cancer, Sema3E was found to be expressed at higher levels compared to normal tissue; PlexinD1 staining too was found to be higher in tumors compared to benign tissue [8]. It was further observed that the cleaved form of Sema3E, Sema3E-p61, was expressed in prostate cancer cell lines. However, the mechanisms regulating cell migration downstream of PlexinD1 in this system are still unclear. On a separate note, Notch signaling ligand—Jag1—has been independently reported to be a marker of poor prostate cancer progression [16]. Consistently, Jagged1-Notch1 signaling has been shown to regulate migration and invasion of prostate cancer cells via the activation of Akt, mTOR and NF-kB signaling pathways [17].

Here we report that the expression of Sema3E-receptor PlexinD1 is under control of the Notch pathway, which is commonly activated in tumors and associated with malignant progression. PlexinD1 regulation occurs at the promoter level, through the canonical Notch signaling pathway mediated by RBPjk/CBF1 transcription factor. Notch signaling cascade has been found to promote cancer metastasis via transcriptional programs regulating cell-cell contact and cell migration [18,19]. Here we show that the upregulation of Notch-target PlexinD1 in prostate cancer cells upholds Slug expression and downregulates E-cadherin levels, a program associated with cell migration, epithelial—to—mesenchymal transition and metastasis.

## Materials and Methods

### Cell lines and chemicals

Tumor cell lines were obtained from ATCC and cultured in a humidified incubator with 5% CO_2_ at 37°C in media supplemented with 10% FBS, penicillin and streptomycin. U87MG, U251, A549 were cultured in DMEM; PC3 and DU145 were cultured in RPMI; SKOV3 was cultured in McCoy media; HUVEC were cultured in EGM-2 media containing growth factors (Clonetics), 100 IU/ml of penicillin G sodium and 100ug/ml of streptomycin sulfate. Cell treatments with gamma secretase inhibitors DAPT (Sigma, D5942-5MG; used 25μM) and RO4929097 (Selleckchem, S1575; used 25μM) were done for 72 hours in media supplemented with 10% FBS. Cell treatments with LY294002 (10μM) and PD98059 (10μM) were done for 24 hours in media supplemented with 10% FBS.

### Gene transfer and RNA interference in mammalian cells

Stable cDNA and shRNA expression in mammalian cells was achieved by means of lentiviral vectors, as previously described [20]. Briefly, calcium phosphate method was used to co-transfect transfer plasmids, packaging vectors and constructs expressing VSV-G envelope protein in 293T cells. Tumor cells were transduced by incubation with lentiviral vector suspensions, in the presence of 8μg/ml polybrene, for 8–12 hours. In other experiments, cDNA and siRNA-expressing constructs were transiently transfected with Lipofectamine 2000 (Life Technologies) according to manufacturer’s instructions.

### siRNA, shRNA and DNA constructs

Notch1 downregulation was achieved by lentiviral-mediated stable expression of (puromycin selectable) shRNA expression constructs: TRCN0000350330, TRCN0000003359 or TRCN0000003362 (indicated as shNotch1_2, shNotch1_3, and shNotch1_5, respectively in the validation experiment shown in S3C Fig); the latter construct was also simply indicated as shNotch1 in experiments throughout the manuscript. PlexinD1 downregulation was achieved by (puromycin selectable) lentiviral-mediated stable expression of shRNA TRCN0000061548 (#48; mainly used in this study and usually dubbed as “shPlexinD1” in the manuscript) and TRCN0000061552 (#52); or by transfection of siRNAs targeting the 3’-UTR sequence of *PLXND1* transcript: GCUACUUGAUCUUGCUGAA. Sema3E knock down was achieved by stable expression of shSema3E sequence: GGTTACGCCTGTCACATAA [5]. Lentiviral vectors were also used to transfer stable expression of VSV-G tagged PlexinD1 (simply dubbed as “PlexinD1” in the manuscript), as previously shown [5]. Constitutively active intracellular domains of Notch1 and Notch3 (N-ICD), or truncated Notch1-ΔE construct [21] (a gift from Claudio Talora and Isabella Screpanti, Rome) were overexpressed by transient transfection. Mouse Jag1 Fc and Dll1 Fc cloned in pTracer CMV vectors [22] were gifts from Takayasu Kato. Hes1-Luc reporter construct, cloned in pGL2 [23] was a gift from Alain Israel, France. 12XCBF/12XCSL-DsRedExpressDR reporter construct was a gift from Federico Bussolino and Urban Lendhal [24]. Slug (*SNAI2* gene) silencing was achieved by transfection of siRNA sequence: 5′- CAAUAAGACCUAUUCAACU-3 [25].

### Luciferase reporter assays

Luciferase reporter assays were done with Hes1 promoter driving firefly luciferase or with a 1567bp region of PlexinD1 promoter (from 1388bp upstream of TSS to 179bp downstream of it) (Genecopoeia) driving secretable Gaussia luciferase. Cells were transfected using lipofectamine 2000 with 1ug of reporter construct in combination with 10-100ng of Notch1-intracellular domain (N1-ICD), N3-ICD, RBPjk1, or DN-RBPjk expressing constructs; a GFP-expressing plasmid was added in all conditions, for internal normalization purposes. 48hrs after transfection, cells were lysed and luciferase assays conducted using luciferase assay system (Promega, Madison, WI, USA). For Hes1-Luc assay, Promega luciferase assay system was used; for PlexinD1-Luc assay, Secrete-Pair Gaussia Luciferase assay system (Genecopoeia) was used. Two mutants of PlexinD1 Gaussia Luc reporter were generated using restriction site-based deletion: Mut1_D1 was deleted between EcoR1 and AfeI sites in the promoter sequence, while Mut2_D1 derived from deletion of the sequence comprised between two NheI sites (both constructs were verified by sequencing).

### Transwell migration assays

Transwell migration assays were performed using Transwell^®^ chamber inserts (Costar, Cambridge, MA) with a porous polycarbonate membrane (8 μM pore size; Corning Costar Incorporated, NY, USA). Briefly, the lower side of the filter was coated with 10 μg/ml fibronectin and blocked with 1% BSA. Approximately 5x10^4^ cells were added in the upper chamber, and allowed to migrate through the filter towards the lower chamber containing the indicated factors. In parallel, the equal number of cells were seeded in cell culture multiwell plates to check for equal cell loading. After 24hrs non-migrated cells from the upper side were removed by a cotton swab, followed by fixing of cells with 11% Glutaraldehyde and staining with crystal violet. Microscopic images were then quantified either by cell counting or by converting to a binary image and quantifying the integrated pixel values using ImageJ (NIH). Experiments were repeated at least twice in replicates, showing consistent results.

### Fluorescence labeling of living cells

Fluorescent labeling of cells was done using Vybrant DiD cell labeling solution (Molecular Probes). Cells were washed with PBS and incubated with 10μM Vybrant DiD in 10% FBS RPMI media for 30 mins, allowing the cells to get labelled. Cells were then trypsinized, centrifuged and washed twice with PBS. Cells were then counted and suspended in PBS and used in extravasation assay. 1 million cells were used per mice.

### Wound healing assay

Wound healing assay was performed in confluent monolayer of cells grown in 6-well plates. A pipette tip was used to make three scratches in cell monolayers; cells were washed twice and images were taken at starting time point, followed by incubation in appropriate media. Later images were taken after 24 hrs, images were aligned and analyzed to score for wound closure (based on measurement of residual wound area). In the case of DAPT and RO4929097 treatments, cells were pre-incubated with the drugs for 48hrs.

### Real time quantitative PCR analysis of gene expression

Total RNA from tumor cell lines was isolated using RNeasy Protect Mini kit (Qiagen) according to the manufacturer's instructions. cDNA preparation was performed according to standard procedures, using M-MLV Reverse Transcriptase (Promega) and oligo-dT primers / random hexamers. PCR was performed by applying Taqman probes (Applied biosystems): PlexinD1 (Hs00391129_m1, Applied biosystems), Notch1 (Hs01062014_m1), Hes1 (Hs00172878_m1), beta actin (Hs99999903_m1). Alternatively, PCR was conducted with SYBR Green Master Mix (Life Technologies) and run in Applied Biosystems 7900HT Fast Real-Time PCR system, by applying the following primer pairs: PlexinD1 (Fwd—ACCGAGCAGTGGATGATTCT, Rev—TCCTGGTGAACGACACAGAC), GAPDH (Fwd—GAAGGTGAAGGTCGGAGTC, Rev—GAAGATGGTGATGGGATTTC), PlexinB1 (Fwd—CACTGAACCCCACACCTTTC, Rev—ATAGCCACCACCTCCTCCTT), Snail (Fwd—GACTACCGCTGCTCCATTCCA, Rev—TCCTCTTCATCACTAATGGGGCTTT), Zeb2 (Fwd—TTCCATTGCTGTGGGCCT, Rev—TTGTGGGAGGGTTACTGTTGG), Slug (Fwd—AGATGCATATTCGGACCCAC, Rev—CCTCATGTTTGTGCAGGAGA), Zeb1 (Fwd—AGCAGTGAAAGAGAAGGGAATGC, Rev—GGTCCTCTTCAGGTGCCTCAG), Twist1 (Fwd—TGTCCGCGTCCCACTAGC, Rev—TGTCCATTTTCTCCTTCTCTGG).

### Metastatic cell extravasation assay

Metastatic tumor cells were labelled in culture by incubation with Vybrant DiD (Life Technologies), according to manufacturer’s specification. One million fluorescent-labelled PC3 cells were then injected into the lateral tail vein of 6–8 weeks old NOD/SCID mice (Charles River Laboratory); experimental groups included 5 animals each. The mice were sacrificed 48 hours after injection, and quantification of metastatic cells in the lungs was done by fluorescence microscopy by analyzing at least four independent microscopic fields per lung, using ImageJ software (NIH) to measure signal intensity. Mice were housed in individual ventilated cages. Animal handling was performed according to international guidelines for animal experimentation and the specific Italian Legislative Decrees no. 116 (27 Jan 1992) and no. 26 (4 Mar 2014). This research project and relative experimental protocols have been approved by the Ethical Committee of the University of Torino and the Ethical Committee for Animal Experimentation at the Institute for Cancer Research and Treatment IRCCS-Candiolo and by the Italian Ministry of Health.

### Protein analysis

Total cell lysates were prepared in a Tris pH 6.8–10% SDS solution (1:1) by heating at 95° for 20 mins. Protein concentration was measured using Pierce BCA protein assay kit as per the company instructions. For western blotting 10–40 ug of protein was resolved on 7.5% or 10% mini gels from Biorad, transferred to nitrocellulose membrane using semi-dry method and immunoblotted. 10% BSA was used for filter blocking in all conditions.

The following primary antibodies were used: against Notch1, Slug and Actin (Santa Cruz Biotechnology), against PlexinD1 (R&D Systems), against Vinculin (Sigma), against Notch3 (Cell Signaling technology), against E-cadherin (BD Transduction laboratories).

### Dataset analysis

The portal assembled by the www.cbioportal.org [26] was used to analyze the correlation of expression of PlexinD1 with Notch signaling genes. We elaborated correlation graphs of prostate adenocarcinoma, colon and rectum adenocarcinoma, thyroid carcinoma, kidney renal clear cell carcinoma TCGA datasets (provisional); mRNA expression z-score threshold was set at +/- 2 fold, and the category of ‘All tumors’ was chosen with input of *PLXND1*, *NOTCH1*, *NOTCH3* and *SNAI2*. Plots of correlation were generated under the heading of 'Plots' —> 'two genes'. The heading of 'Mutual exclusivity' gave the p values, while the input of individual genes yielded the spearman correlation coefficient under the heading 'co-expression'. GEO—GSE54460 prostate cancer cell dataset was also analyzed for correlation between *SNAI2* and *PLXND1* genes, while multiple genes expression fold change (vs. control) were analyzed in GEO-GSE40403 endothelial cell dataset, upon Jag1 overexpression or Notch1 knock down.

### Statistical analysis

All in vitro and in vivo experiments were performed at least two-three times. Representative results of qPCR experiments are shown. T-test was used to assess statistical significance, indicated in graphs as follows: *** (p<0.001), ** (p<0.01), * (p≤0.05).

## Results

### PlexinD1 is a novel transcriptional target of Notch signaling

PlexinD1 overexpression in high-grade vs. normal tissues or low-grade human tumors has been reported in a number of studies [5,6,8]. Previous data suggested that PlexinD1 may be induced in the vasculature in response to hypoxia or Vascular Endothelial Growth Factor (VEGF) [11,12], conditions which are also known to regulate cancer cells. In preliminary experiments we found that VEGF significantly induced *PLXND1* mRNA expression in endothelial HUVEC cells, but not in a range of human carcinoma cells; moreover, we also failed to observe any significant upregulation of PlexinD1 levels in cancer cell lines exposed to low oxygen tensions (data not shown). These data suggested that PlexinD1 expression in tumor cells may be regulated by mechanisms different from those observed in endothelial cells.

**Fig 1:**
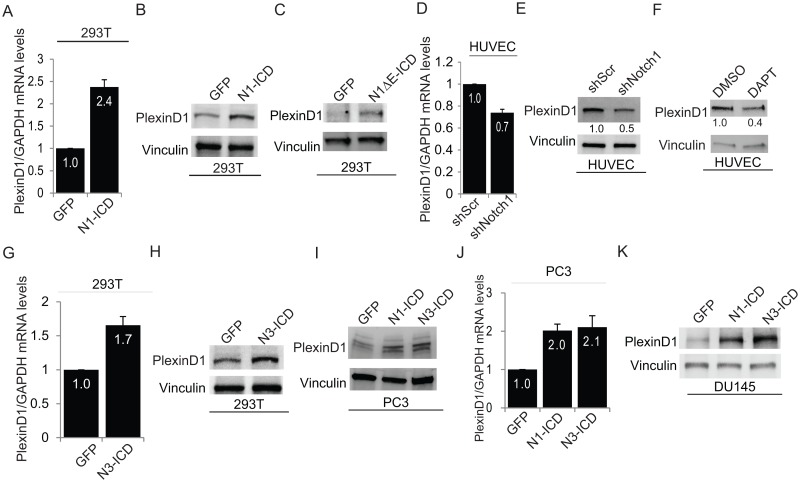
PlexinD1 is a putative Notch signaling target. (A, B) HEK-293T cells were transiently transfected with a constitutively activated Notch-intracellular domain construct (N1-ICD) or with GFP expressing construct. PlexinD1 mRNA levels were measured by qPCR after 72 hrs (A), and protein lysates were analyzed by immunoblotting for PlexinD1 and vinculin (B). (C) HEK-293T cells were transiently transfected with Notch1 ΔE-ICD and cell lysates were immunoblotted for PlexinD1 and vinculin, as above. (D, E) HUVEC endothelial cells were transduced to stably express Notch1-targeted shRNAs (shNotch1); mRNA levels were analyzed by qPCR (D), and protein lysates by immunoblotting (E; relative PlexinD1 band intensity to vinculin levels was quantified and normalized to controls), as above. (F) HUVEC cells were treated with gamma-secretase inhibitor DAPT (25μM) to block Notch signaling, or DMSO (vehicle), for 72 hrs; lysates were analyzed by immunoblotting for PlexinD1 and vinculin (relative PlexinD1 band intensity to vinculin levels was quantified and normalized to controls). (G, H) HEK-293T cells were transiently transfected with activated Notch3 intracellular domain (N3-ICD); after 72hrs, mRNA levels (G) and protein lysates (H) were analyzed, as above. (I, J) PC3 prostate cancer cells transiently transfected with N1-ICD or N3-ICD were analyzed for expression of PlexinD1 by qPCR (I) or western blotting (J). (K) DU145 prostate cancer cells transiently transfected with N1-ICD and N3-ICD were analyzed by immunoblotting for PlexinD1 and vinculin, as above. Mean values ± SD are shown in all graphs; relative gene expression levels were normalized to controls.

Interestingly, by data mining in gene expression datasets, we found that—among other genes–*PLXND1* levels were induced almost 4-fold upon constitutive Notch1 activation in glioblastoma stem cells [27]. Initially, we validated these raw data in two glioblastoma cell lines—U87MG and U251. Indeed, upon overexpressing a constitutively activated form of Notch1 (N1-ICD) we observed an appreciable increase of PlexinD1 protein levels (S1A and S1B Fig). These findings in glioblastoma cell lines encouraged us to further study this novel regulatory mechanism in other cell types. Constitutive Notch signaling established by transfection of N1-ICD or a ligand-independent activatable receptor (Notch1—ΔE ICD [28]), significantly induced endogenous PlexinD1 mRNA and protein levels in 293T cells (Fig 1A and 1C). In other experiments, we analyzed HUVEC endothelial cells, which abundantly express PlexinD1 in basal conditions, and subjected them to stable Notch1 knock down by shRNAs or blunted its signaling cascade with the gamma secretase inhibitor DAPT [29]. In either case, interfering with the Notch pathway led to concomitant PlexinD1 downregulation at mRNA and protein level (Fig 1D and 1F); conversely, NICD overexpression further induced PlexinD1 levels (S1C Fig). These data are consistent with genome-wide expression analysis in endothelial cells (GEO Dataset—GSE40403) upon overexpression of the Notch ligand Jagged1 (S1D Fig) or Notch1 gene silencing (S1E Fig), as in either case PlexinD1 levels were concordantly regulated with those of Notch transcriptional targets HES1 and HEY1. Together, these data supported the conclusion that Notch signaling can positively regulate PlexinD1 mRNA and protein expression in normal and tumor cells.

Prompted by these findings, we analyzed the TCGA dataset of different human cancers to search any potential correlation between PlexinD1 expression and that of Notch family members. To this end, we determined the correlation in the expression of PlexinD1 with Notch1 and Notch3 in datasets of prostate, colon, thyroid and kidney cancers. As shown in S2A Fig, PlexinD1 showed a positive Spearman correlation coefficient with Notch1 levels, ranging from 0.43 (prostate cancer) to 0.75 (kidney renal clear cell carcinoma). Notably, Notch signaling is implicated in the invasiveness of prostate carcinoma cells, and its expression correlates with tumor grade [30]; moreover, Blanc et al. independently reported that PlexinD1 is upregulated in prostate cancer [8].

Another Notch family receptor, Notch3, displayed significant positive Spearman correlation with PlexinD1 in different tumor types (S2B Fig). As expected, the constitutively active intracellular domains of both genes were similarly capable of activating the transcription of known target genes (S2C and S2D Fig). Notably, constitutively active Notch3-ICD also upregulated PlexinD1 levels (Fig 1G and 1H), as seen for N1-ICD. Moreover, both Notch1 and Notch3 induced PlexinD1 expression in two prostate cancer cell lines PC3 and DU145 (Fig 1I, 1J and 1K) and in U87MG (data not shown). Altogether, our data strongly indicated that Notch signaling is sufficient and required to upregulate PlexinD1 expression in different cellular models.

### Notch regulates *PLXND1* gene promoter via the canonical RBPjk-dependent pathway

**Fig 2:**
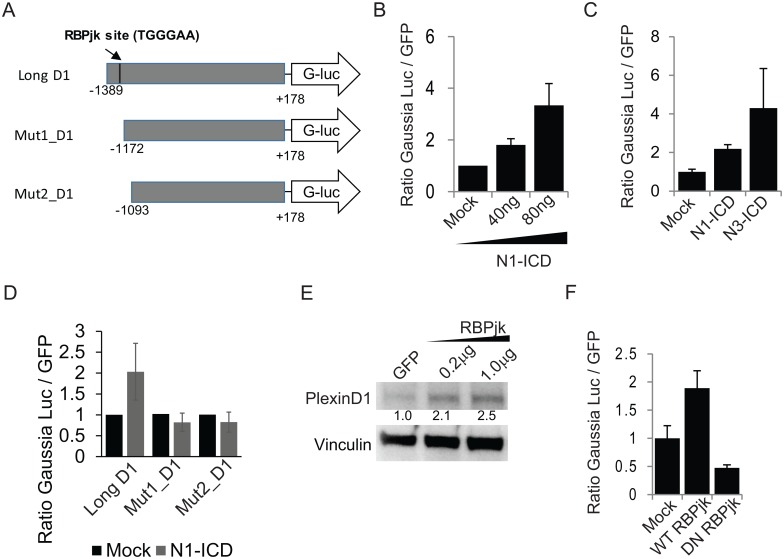
Notch signaling upregulates PlexinD1 promoter activity. (A) Schematic of the reporter construct pEZX-PGO2, containing a 1567 bp region from *PLXND1* gene promoter (position 1 being transcription start) fused to *Gaussia* luciferase reporter cassette, and of two mutant constructs with deletions of RBPjk binding site (positioned at -1382 bp upstream transcription start). (B) *PLXND1* promoter-induced *Gaussia* luciferase activity was revealed in cell-conditioned media of 293T cells co-transfected with mock plasmid, or with N1-ICD (indicated amounts), in combination with the reporter construct. (C) 293T cells were transiently co-transfected with either mock plasmid, N1-ICD or N3-ICD in combination with PlexinD1 promoter reporter construct. (D) Luciferase reporter activity was tested in 293T cells transiently transfected with N1-ICD (or mock), in association with *PLXND1* promoter region (Long D1) or with mutated promoter constructs lacking RBPjk binding sites (Mut1_D1 and Mut2_D1, described in panel (A). (E) 293T cells were transiently transfected with the indicated amounts of RBPjk cDNA; cell lysates were analyzed by immunoblotting for PlexinD1 and vinculin; relative band intensity was quantified and normalized to control. (F) 293T cells were transiently co-transfected with wild-type RBPjk or dominant-negative RBPjk (or mock) in combination with *PLXND1* promoter reporter construct, and luciferase activity was revealed in cell-conditioned media, as in (B). Bar graphs indicate mean values (normalized to controls) ± normalized SD.

Transcriptional gene regulation by Notch family members may be mediated by a so-called canonical pathway, dependent on the association of nuclear-translocated NICD fragment with the DNA-binding transcriptional effector molecule RBPjk/CBF1, or by alternative non-canonical pathways [31]. Notably, bioinformatic analysis revealed the presence of a putative RBPjk binding site in PlexinD1 promoter sequence (Fig 2A). Thus, we assayed whether Notch1 signaling could control *PLXND1* gene promoter activity by using a reporter construct containing a 1567 bp region upstream *PLXND1* transcription start site and driving the expression of secretable Gaussia luciferase (shown in Fig 2A). We co-transfected the reporter construct in association with increasing amounts of N1-ICD, and found that Notch1 signaling enhanced *PLXND1* promoter activity in a dose dependent manner (Fig 2B). In the same experimental setting, constitutive active Notch-3 also elicited similar effects as Notch-1 (Fig 2C), confirming that both receptors can regulate *PLXND1* promoter activity.

In order to validate the relevance of the putative RBPjk binding site in *PLXND1* promoter, we assayed two truncated reporter constructs lacking this sequence (sketched in Fig 2A), and found that they were insensitive to transcriptional regulation by N1-ICD (Fig 2D). Further consistent with these data, upon ectopic expression of RBPjk transcriptional effector in cells we observed a significant increase in endogenous PlexinD1 levels (Fig 2E), and enhanced *PLXND1* promoter activity was observed in a reporter assay (Fig 2F). In contrast, a dominant negative form of RBPjk that associates with NICD but it is unable to bind DNA (DN-RBPjk [32]) significantly reduced basal *PLXND1* promoter activity (Fig 2F), leading to the conclusion that PlexinD1 expression is upregulated by the association of Notch-ICD with its transcriptional effector RBPjk.

### Notch signaling is responsible for sustaining PlexinD1 expression in cancer cells

**Fig 3:**
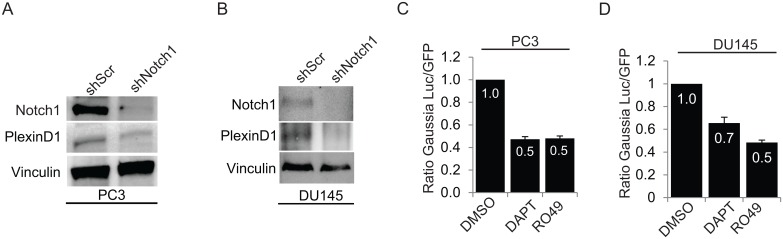
Downregulation of Notch signaling inhibits PlexinD1 expression. (A) PC3 stably expressing shNotch1 or scrambled shRNA (shScr) as control were generated, and protein lysates were analyzed by immunoblotting to reveal PlexinD1, Notch1 (intracellular domain, at 110 kDa) and vinculin levels. (B) As above, PlexinD1, Notch1-ICD and vinculin levels were analyzed by immunoblotting in DU145 stably expressing shScr or shNotch1, as in (A). (C, D) PC3 and DU145 cells transfected with *PLXND1* promoter reporter construct (as in Fig 2) were treated with Notch inhibitors DAPT (25μM) and RO4929097 (25μM) for 72 hrs, and *Gaussia* luciferase activity was assessed to reveal *PLXND1* promoter regulation.

In order to know if Notch-dependent regulation of PlexinD1 widely occurs in cancer cells, we stably knocked-down Notch1 expression by shRNA in a range of cell lines derived from colon (KM-20, COLO741), prostate (PC3), lung cancer (A549) and melanoma (MDA435), and consistently observed PlexinD1 downregulation (S3A Fig). Of note, we observed that the regulation of PlexinD1 was specific, as Notch-targeting shRNAs did not affect the expression of another plexin family member implicated in cancer progression—PlexinB1 (S3B Fig). Notch1 signaling appeared to be particularly relevant in regulating PlexinD1 mRNA levels in prostate cancer cells PC3 and DU145, and this was confirmed at the protein level (Fig 3A and 3B). Importantly, we identified three independent shRNA sequences that could efficiently silence PlexinD1 expression in prostate cancer cells (S3C Fig), and found that all of them caused concomitant downregulation of PlexinD1 levels, clearly suggesting a mechanistic link. Notch signaling is basally present in normal and tumor cells, mainly due to autocrine/juxtacrine ligand activity. Inhibitors of gamma-secretases are widely used reversible inhibitors that achieve a compound inhibition of all Notch family members; these drugs include DAPT [29] and RO4929097 [33], which is also under assessment in clinical trials. We confirmed that these drugs could efficiently inhibit Notch signaling in different cell types carrying basal activation of the endogenous molecules, demonstrated by the presence of the intracellular cleaved domain (S4A Fig). Notably, we found that 72-hour treatment with Notch inhibitors is sufficient to downregulate PlexinD1 levels both in endothelial and in prostate cancer cells (S4B and S4D Fig). Moreover, the treatment with DAPT or RO4929097 inhibited *PLXND1* promoter activity in PC3 and DU145 cells (Fig 3C and 3D). Further experiments demonstrated that Notch inhibitors can downregulate PlexinD1 expression in additional cancer cells of different histotype (S4E Fig). These data establish the concept that PlexinD1 expression is under control of Notch signaling in prostate cancer cells, as well as in other tumor cell types.

Notch signaling is activated in response to different members of the Notch-ligand family. For instance, the membrane-bound ligand Jag1 is overexpressed in human tumors and has been linked to metastasis [16]. Thus we tested the activity of a Jag-derived soluble peptide capable of activating Notch signaling in cellular models [34]. Interestingly, Jag1 peptide could similarly induce the expression of the canonical Notch target Hes1, as well as that of PlexinD1 in PC3 cells (S5A Fig). Also, PlexinD1 promoter reporter activity was upregulated by Jag1 peptide, while it was completely blunted by the combined treatment with gamma secretase inhibitor RO4929097 (S5B Fig). In another set of experiments, we transiently overexpressed either mouse Jag1-Fc or another Notch ligand relevant in cancer, mouse Dll1-Fc [35,36] in PC3 cells. Again, both Notch ligands induced PlexinD1 expression (S5C Fig), as well as PlexinD1 promoter activity (S5D Fig), although to a lower extent compared to constitutive ligand-independent receptor activation.

### Notch and PlexinD1 signaling controls prostate cancer cell migration

**Fig 4:**
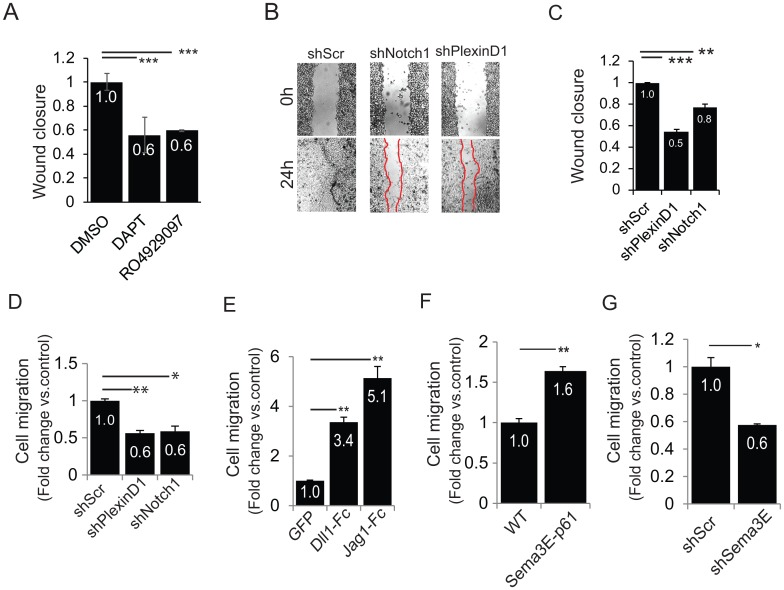
Notch and PlexinD1 signaling regulate prostate cancer cell migration. (A) Wound healing assay in PC3 cells treated with gamma secretase inhibitors—DAPT and RO4929097 (as in Fig 3C). Wound closure (24 hours from scratch) was quantified relative to wound width at start time. (B, C) The migration of PC3 cells stably expressing shScr, shNotch1, shPlexinD1 was analyzed in wound healing assays, as above. Panel B shows the quantification of wound sizes at the end of the experiment. (D) The migration of PC3 cells stably expressing shScr, shPlexinD1 and shNotch1 was assayed in overnight Boyden chamber experiments with transwell inserts. Bar graphs indicate mean values ± SD (normalized to controls). (E) PC3 cells overexpressing Dll1-Fc and Jag1-Fc were analyzed in Boyden chamber experiment, as above. (F) PC3 cells transduced to express an autocrine p61-Sema3E circuit were analyzed in Boyden chamber experiments, as above. (G) PC3 cells stably expressing shSema3E compared to shScr were analyzed in Boyden chamber experiments, as above. Mean ± SD is shown in all graphs; values were normalized to respective controls.

A number of studies have reported that upregulated Notch signaling is associated with invasion and metastasis in prostate cancer [37,38]. Notably, PlexinD1 levels are similarly regulated in human prostate tumors and this signaling cascade controls prostate cancer cell behavior [8]; however, the underlying molecular mechanisms were not elucidated. In order to assess the functional relevance of Notch-PlexinD1 signaling axis in prostate cancer cells, we performed wound healing and Boyden chamber transwell migration assays with PC3 and DU145 prostate cancer cells. We tested the effect of gamma secretase inhibitors blocking pan-Notch signaling on PC3 migration, and consistent with previous data on DAPT treatment [29] we observed a significant reduction of migratory ability (Fig 4A). Then, we observed that both Notch1 and PlexinD1 silencing by shRNAs led to decreased migration in wound healing assays (Fig 4B and 4C). Moreover, knocking down either Notch1 or PlexinD1 similarly decreased the migration of PC3 and DU145 prostate cancer cells in transwell migration assays (Fig 4D and S6A and S6B Fig). The role of PlexinD1 signaling in the regulation of cell migration was further validated upon gene knock down with two independent shRNA sequences (S6C and S6D Fig), as well as by functional rescue upon re-expressing PlexinD1 in silenced cells by means of ectopic cDNA transfection (S6E and S6F Fig). These data are consistent with the notion that these cancer cells express endogenous Notch and PlexinD1 ligands, empowering basal autocrine signaling circuits [8,39].

Indeed, the overexpression of Notch ligands Dll1-Fc and Jag1-Fc in PC3 cells led to significant induction of cell migration (Fig 4E). Moreover, similar results were observed in cells with an enhanced autocrine circuit of the PlexinD1-ligand Sema3E-p61 (Fig 4F), and conversely we observed decreased migration of PC3 cells depleted of endogenous Sema3E (Fig 4G). Altogether these experiments indicate that both Notch and PlexinD1 signaling cascades play matching roles in regulating cell migration, consistent with Notch activity upregulating PlexinD1 expression in prostate cancer cells. Yet, the molecular mechanisms by which Sema3E-PlexinD1 signaling could induce the migration of prostate cancer cells are presently unknown.

### Notch and PlexinD1 coordinately regulate gene expression in prostate cancer cells

**Fig 5:**
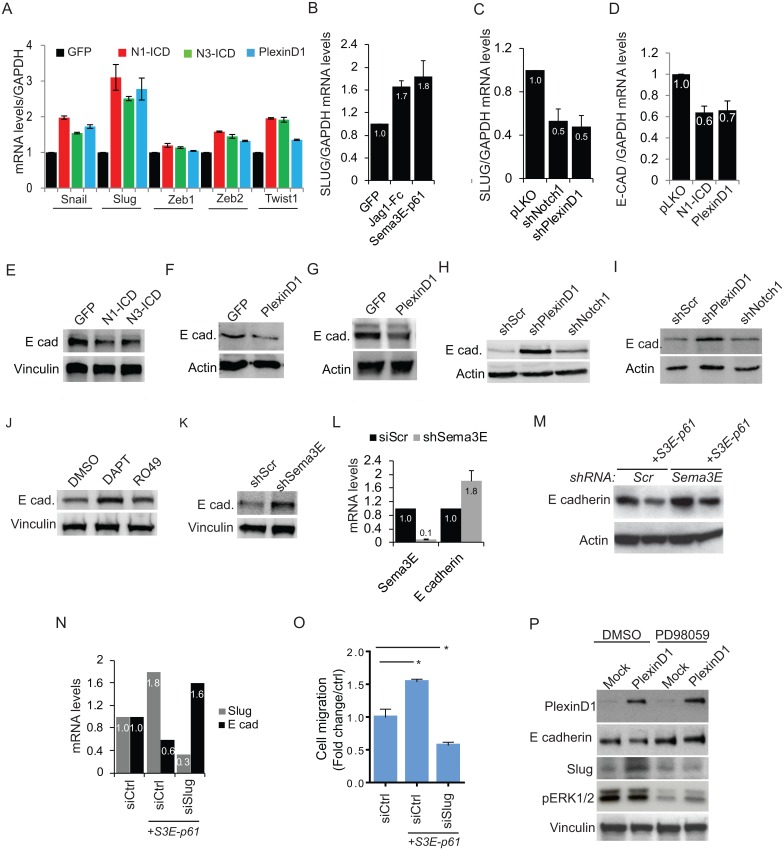
Notch1, Notch3 and PlexinD1 regulate EMT markers in prostate cancer cells. (A) PC3 were transiently transfected with GFP, N1-ICD, N3-ICD or PlexinD1, and the mRNA levels of Snail, Slug, Zeb1, Zeb2, Twist1 were analyzed by qPCR (48 hours later). (B) PC3 cells were transfected with GFP, Jag1-Fc, or Sema3E-p61; Slug mRNA levels were analyzed, as above. (C) Slug mRNA levels were assessed in PC3 cells stably expressing pLKO or silenced for Notch1 and PlexinD1. (D) PC3 transiently transfected with pLKO, N1-ICD and PlexinD1 were analyzed for E-cadherin mRNA levels by qPCR. (E) PC3 cells were transiently transfected with GFP, N1-ICD or N3-ICD; after 48 hrs lysates were analyzed by immunoblotting to reveal E-cadherin levels. (F, G) PC3 and DU145 cells transfected with PlexinD1 were analyzed by immunoblotting to reveal E-cadherin levels. (H, I) PC3 and DU145 cells, respectively, stably expressing shScr, shPlexinD1 and shNotch1 were analyzed by immunoblotting to reveal E-cadherin levels. (J) PC3 cells were treated with DMSO, DAPT (25μM) or RO4929097 (25μM) for 72 hrs and protein lysates were analyzed to reveal E-cadherin levels. (K, L) PC3 cells stably expressing shScr or shSema3E were analyzed by western blotting (K) or qPCR (L) to reveal E-cadherin levels. (M) Analysis of E cadherin levels by immunoblotting in PC3 cells stably silenced for Sema3E, and upon re-expression of Sema3E-p61 (S3E-p61). (N) E cadherin and Slug expression levels assessed by qPCR upon overexpression of Sema3E-p61 in control and Slug-depleted cells (by siRNA). (O) The migration of PC3 cells treated as in previous panels was assessed in Boyden Chamber assays. (P) Immunoblotting analysis of E cadherin levels in PC3 cells transfected to overexpress PlexinD1 (or mock), with or without treatment with MAPK inhibitor PD98059 (10μM). Bar graphs display mean values ± SD.

Notch signaling has been associated with the activation of transcription factors involved in the so-called epithelial-mesenchymal transition (EMT), which promotes cell migration in development, as well as cancer invasion and metastasis [19,40,41,42,43]. Thus, in order to identify the protein mediators downstream to PlexinD1, we analyzed the expression of EMT transcription factors Snail, Slug, Twist, Zeb1 and Zeb2 in PC3 prostate cancer cells. Interestingly, Slug levels were consistently upregulated upon expression of activated Notch1, Notch3 or direct PlexinD1 overexpression (Fig 5A). In addition, both Notch ligand Jag1 and PlexinD1 ligand Sema3E-p61 significantly upregulated Slug levels (Fig 5B); conversely, Slug expression was reduced in cells subjected to Notch1 or PlexinD1 silencing (Fig 5C). Interestingly, gene expression profiling of human prostate cancer samples reported in TCGA database indicates significant correlation between Slug expression and both Notch1 and PlexinD1 levels (S7A Fig); moreover, similar results were obtained upon analyzing a distinct dataset (S7B Fig).

E-cadherin downregulation is a hallmark associated with the activity of various transcription factors driving the EMT program, including Slug. Indeed, reduced E-cadherin levels are strongly linked to a gain of metastatic ability of prostate cancer cells to secondary sites [44,45]. Notably, overexpression of Notch1, Notch3, or PlexinD1 downregulated E-cadherin levels in prostate cancer cells (Fig 5D, 5E, 5F and 5G). Conversely, cells knocked down for PlexinD1, Sema3E or Notch1 expression, or treated with gamma secretase inhibitors of Notch signaling, underwent E-cadherin upregulation (Fig 5H, 5I and 5L).

The specific requirement for Sema3E/PlexinD1 signaling to regulate E-cadherin levels was further indicated by the functional rescue upon re-expression of a (non RNAi-sensitive) active form of the ligand p61-Sema3E (Fig 5M). Moreover, similar results were obtained by transfecting PlexinD1 cDNA in gene silenced cells (S8A Fig). Interestingly, a point mutant of PlexinD1 known to lack GAP activity (RA-mutant; [4] was still fully competent to mediate E-cadherin downregulation (S8B Fig), consistent with the idea that PlexinD1 can mediate multiple signaling cascades in cancer cells [46]. Furthermore, we found that E-cadherin upregulation in cancer cells subjected to PlexinD1 knock down was maintained upon transplantation in mice and tumor formation *in vivo* (S9A and S9B Fig).

In further experiments we confirmed the relevant role of Slug to mediate Sema3E-induced E-cadherin downregulation (Fig 5N) and increased cancer cell migration (Fig 5O). Notably, it was previously reported that the nuclear translocation of another transcription factor (Snail) induced by Sema3E in ovarian cancer cells was mediated by the PI3K/AKT pathway [6]. Thus we tested whether inhibitors of intracellular signaling effectors PI3K and MAPK could interfere with Sema3E/PlexinD1 dependent upregulation of Slug levels in prostate cancer cells. Intriguingly, while we could not detect any change in presence of 10μM LY294002 PI3K inhibitor (not shown), PlexinD1-dependent Slug upregulation (and consequent E-cadherin suppression) was blunted upon treatment with the MAPK inhibitor PD98059 (10μM) (Fig 5P).

Taken together, these data indicate that both Notch and PlexinD1 signaling can upregulate Slug expression in prostate cancer cells and concomitantly downregulate E-cadherin expression. In keeping with its role as transcriptional target of Notch, PlexinD1 could therefore also be envisaged as relevant effector of Notch signaling cascade.

### PlexinD1 acts downstream to Notch signaling to empower cancer cell migration

**Fig 6:**
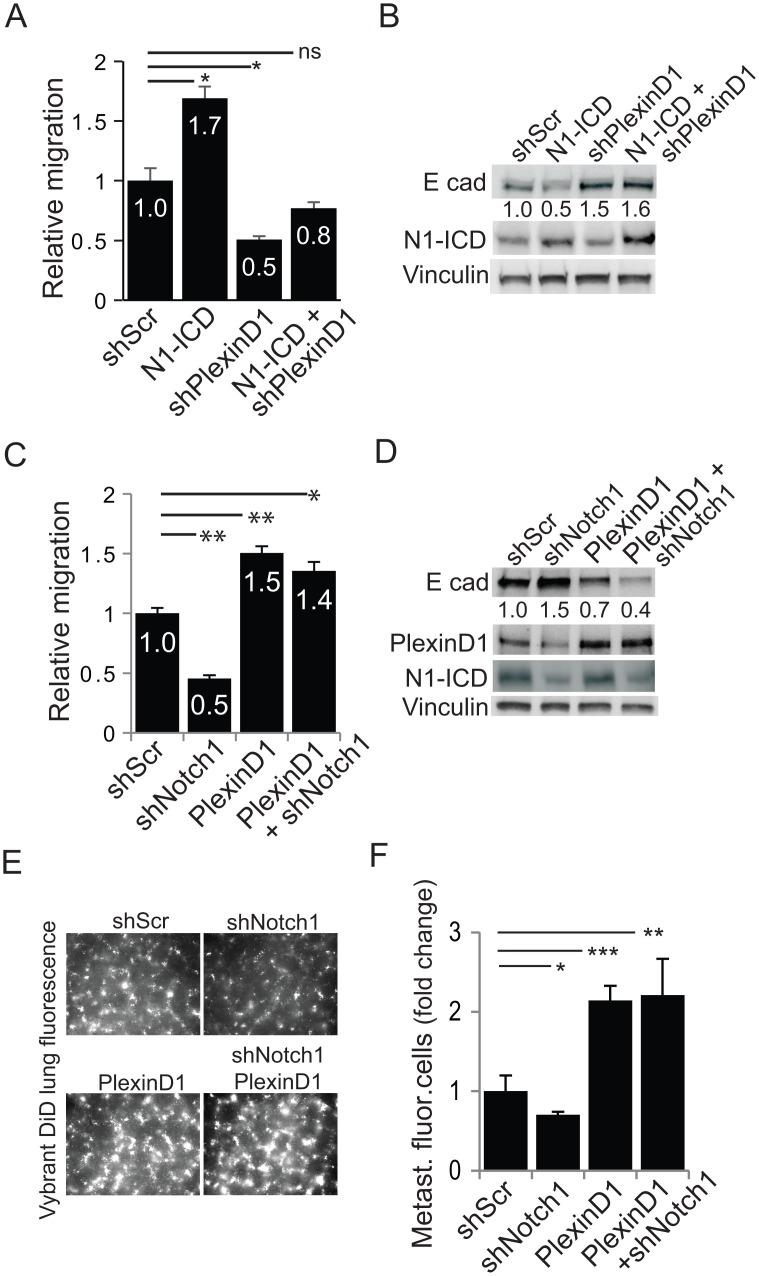
PlexinD1 acts downstream of Notch signaling in down-regulating E-cadherin and promoting cancer cell migration. (A, B) PC3 cells were transiently transfected with either shScr, N1-ICD, shPlexinD1, or a combination of N1-ICD and shPlexinD1. 72 hrs after transfection, cell migration was analysed by Boyden chamber assays (A). Moreover, cell lysates were analyzed by immunoblotting to reveal E-cadherin, Notch1-ICD and vinculin levels (B); relative E-cadherin band intensity to vinculin levels was quantified and normalized to controls. (C, D) PC3 cells were transiently transfected with either shScr, shNotch1, PlexinD1, or a combination of shNotch1 and PlexinD1. 72 hrs after transfection, cell migration was analysed by Boyden chamber assays (C). Moreover, cell lysates were analyzed by immunoblotting to reveal E-cadherin, N1-ICD, PlexinD1 and vinculin levels (D) and relative E-cadherin band intensity to vinculin levels was quantified and normalized to controls. (E, F) PC3 cells transfected as in C-D were analyzed in vivo by metastatic extravasation assay upon tail vein injection in mice. Representative images are shown (E) along with fluorescence intensity quantification (F). Bar graphs show mean values ± SD (normalized to control).

As discussed above, transcriptional programs downstream to Notch signaling are known to foster cancer cell migration/invasion [47,48,49]. Indeed, our data indicate that N1-ICD concomitantly promoted prostate cancer cells migration and downregulated E-cadherin levels. As this also correlated with increased PlexinD1 expression, we asked whether PlexinD1 could be the driver eliciting downstream regulatory mechanisms and cell migration. Thereby we transfected constitutively active Notch in cells carrying PlexinD1-targeted shRNAs; in this setting, PlexinD1 levels could not be induced by N1-ICD. Importantly, this prevented E-cadherin downregulation and a gain of cancer cell migration in response to constitutive Notch signaling (Fig 6A and 6B). These data strongly suggest that PlexinD1 is required to mediate Notch activity in controlling cell migration.

We also performed the complementary experiment: since Notch1 silencing caused reduced migration of PC3 and DU145 cells, correlating with lower PlexinD1 levels, we asked whether the forced expression of PlexinD1 could restore, at least in part, this functional loss. As shown in Fig 6C and 6D, PlexinD1 overexpression was able to downregulate E-cadherin levels independent of Notch, and fully rescued migration defect (and E-cadherin loss) associated with reduced Notch1 activity, consistent with the idea that PlexinD1 is located downstream of Notch in the signaling cascade leading to E-cadherin regulation and increased cell migration. Importantly, this effect was also appreciable in *in vivo* setting, where PlexinD1 overexpression promoted metastatic cancer cell extravasation independently of Notch1 (Fig 6E and 6F).

## Discussion

Previous studies have shown that Sema3E-PlexinD1 signaling is deregulated in human cancers; however, the molecular mechanisms responsible for PlexinD1 overexpression remained undetermined. According to our findings, canonical Notch signaling is a positive regulator of PlexinD1 expression in an RBPjk dependent manner. This is supported by experimental evidence in a range of cells of different tumor origin, either subjected to Notch1 genetic silencing or treated with gamma secretase inhibitors to block this signaling cascade. Furthermore, we demonstrated the positive regulatory activity of Notch signaling on *PLXND1* promoter, dependent on the canonical RBPJ-kappa pathway. Moreover, our data suggest that Notch1 and Notch3 may act redundantly in PlexinD1 regulation. Notably, Notch signaling is known to be upregulated during progression of colon, breast, ovarian and prostate cancers [16,50,51]. Independently, it was shown that PlexinD1 is overexpressed in the same tumor types [5,6,7,8]. Indeed, by TCGA data mining we found a positive correlation of *NOTCH1* and *NOTCH3* expression with *PLXND1*, supporting our hypothesis. In apparent conflict with this, a negative crosstalk has been observed in retinal vessel development where Notch signaling was found to downregulate PlexinD1[11]. However, PlexinD1 downregulation in zebrafish Notch signaling mutants has also been reported [52], which supports our data in human cells.

The functional role of PlexinD1 signaling in cancer cell migration is under investigation. For instance, Sema3E revealed dual regulatory activity on migration. A recombinant molecule corresponding to the precursor full-length Sema3E may display inhibitory function [8,53]. However, cancer cells often carry an autocrine loop of proteolytically processed isoform of Sema3E (dubbed p61-Sema3E) which instead promotes cancer cell migration in PlexinD1-dependent manner [5]. Indeed, here we show that p61-Sema3E signaling enhanced prostate cancer cells migration, while knocking down the endogenous Sema3E or PlexinD1 levels inhibited it. Previous work has reported that PlexinD1 can functionally interact with ErbB2 and elicit invasion and metastasis [5]. However, we found that this mechanism is unlikely to have a role in PC3 prostate cancer cells, as they express low levels of ErbB2 and the treatment with the kinase inhibitor lapatinib didn’t seem to interfere with Sema3E/PlexinD1-mediated effects (M.R., unpublished results). In ovarian cancer cells it was reported that Sema3E signaling drives nuclear translocation of Snail, a transcription factor controlling E-cadherin expression. Our work now demonstrates that PlexinD1 regulates E-cadherin levels and cell migration in prostate cancer cells. Intriguingly, we could not detect any significant change of subcellular Snail localization upon N1-ICD or PlexinD1 overexpression in prostate cancer cells (data not shown), but we observed significant induction in the expression of Slug, another transcription factor known to drive E-cadherin loss. Notably, Slug was recently reported to be required for EMT and invasiveness of breast cancer cells in response to Notch signaling [49]. Thus, based on our findings, we propose that in prostate cancer cells PlexinD1 mediates Slug upregulation elicited by Notch signaling. Interestingly, the molecular mechanisms linking Sema3E/PlexinD1 to Slug upregulation seem to be distinct from downstream pathways reported so far, and putatively implicate MAPK activity; further studies are warranted to elucidate this novel signaling cascade.

## Conclusion

In this study we have shown for the first time that Notch signaling (both Notch1 and Notch3 receptors) upregulates PlexinD1 expression at the promoter level in different cells. This regulatory mechanism is dependent on canonical Notch signaling, as RBPjk itself can induce PlexinD1 expression, while this is blocked by its dominant negative form. Notch1 and Notch3 expression is positively correlated with that of PlexinD1 in patient cancer samples. We have revealed that PlexinD1 expression is required to mediate cancer cell migration induced by constitutive Notch signaling in prostate cancer cells; moreover, PlexinD1 expression is sufficient to promote cancer cell migration downstream of Notch. Notably, the overexpression of Notch1, Notch3 and PlexinD1 induces Slug transcription factor and downregulates E-cadherin levels in prostate cancer cells, a phenotype associated with increased cell migration and metastatic potential in vivo. This signaling cascade might be important in the gain of invasive and metastatic ability of human prostate tumors, and its therapeutic targeting could be envisaged by treatment with Notch signaling inhibitors.

## Supporting Information

S1 Fig Notch signaling regulates PlexinD1 expression in cancer and normal cells. (A, B) Constitutively active Notch1-intracellular domain (N1-ICD) was overexpressed in glioblastoma-derived cell lines U87 and U251, and PlexinD1 protein expression was determined in cell lysates by immunoblotting. Vinculin levels were analyzed to provide reference for protein loading. (C) PlexinD1 protein detection by immunoblotting in HUVEC cells overexpressing N1-ICD. (D) GEO dataset GSE40403 was analyzed for expression of *HES1*, *HEY1* and *PLXND1* in HUVEC cells overexpressing Notch ligand Jag1 (D) or subjected to *NOTCH1* knock-down (E).
(PDF)

S2 Fig Correlation of mRNA expression of Notch1/Notch3 and PlexinD1 in different human cancers. (A-B) TCGA datasets of prostate, colon and rectum adenocarcinoma, thyroid and kidney renal cell carcinoma were analyzed using cBioportal interface for Notch1 (or Notch3, respectively) and PlexinD1 expression levels; two-gene correlations were plotted, also indicating Spearman correlation coefficients (r). p value was calculated from the panel of ‘mutual exclusivity and co-occurrence analysis’. (C) 293T cells were transiently transfected with mock plasmid, N1-ICD or N3-ICD in combination with Hes1 Luc reporter plasmid. 48 hrs after transfection, cells were lysed and Hes1-luc reporter activity was measured. Mean values ± SD are shown. (D) COS7 cells were transfected with 12X CBF dsRed reporter in combination with mock plasmid, N1-ICD and N3-ICD. Mean ± SD is shown.
(PDF)

S3 Fig Notch signaling specifically sustains PlexinD1 expression. (A) PlexinD1 mRNA levels were analyzed in KM20, PC3, A549, COLO741, MDA435 cancer cells stably expressing shNotch1 or shScr. Relative gene expression was normalized to control cells. (B) PlexinB1 mRNA levels were analyzed by qPCR in the indicated tumor cells expressing shNotch1 (or shScr). (C) Three independent shRNAs targeting Notch1 were transfected in PC3 cells to validate the specific effect of this knock down on PlexinD1 mRNA levels. Bar graphs show mean values ± SD.
(PDF)

S4 Fig Notch signaling inhibition downregulates PlexinD1 levels. (A) The presence of activated Notch1 intracellular cleaved domain (N1-ICD) in 293T and PC3 cells was revealed by immunoblotting with an isoform specific anti-Val_1744_ antibody; N1-ICD levels dramatically dropped in cells treated with (γ-secretase) Notch cleavage inhibitors DAPT (25μM) or RO4929097 (25μM). (B) The mRNA levels of Notch target genes *HES1* and *PLXND1* were analyzed by qPCR in HUVEC endothelial cells, in basal conditions and upon treatment with Notch inhibitors DAPT or RO4929097. (C-D) PC3 cells were treated with DAPT (25μM) and RO4929097 (25μM) for 72 hrs and *PLXND1* mRNA were analyzed by qPCR (C); independently, protein lysates were analyzed for PlexinD1 and vinculin by immunoblotting (D). (E) MCF7 and KM20 carcinoma cells were treated with Notch inhibitors DAPT or RO4929097 for 72 hrs (in independent experiments), and cell lysates were analyzed by immunoblotting to reveal PlexinD1 expression levels.
(PDF)

S5 Fig Regulation of PlexinD1 expression by Notch ligands. (A) PC3 cells were treated with 7.5 μM Jag1 soluble peptide for 24hrs and compared with untreated control cells. PlexinD1 and Hes1 mRNA levels were analyzed by qPCR. (B) PC3 cells were transfected with PlexinD1 promoter reporter construct (as in main Fig 2); the following day the cells were treated with Jag1 peptide 7.5 μM or Jag1 peptide plus Notch inhibitor RO4929097 (25μM), and after 24hrs cell-conditioned media were analyzed to reveal *Gaussia* luciferase activity. (C) PC3 cells were transiently transfected with either GFP, Dll1-Fc or Jag1-Fc; 48 hours later, PlexinD1 and vinculin levels were analyzed by immunoblotting; relative band intensity was quantified and normalized to controls. (D) PC3 cells were transfected with PlexinD1 promoter reporter construct in combination with Dll1-Fc, Jag1-Fc and N1-ICD. Mean ± SD is shown.
(PDF)

S6 Fig DU145 and PC3 cell migration is regulated by Notch and PlexinD1 signaling. (A) Analysis of DU145 prostate cancer cells migration (in transwell Boyden Chamber assays) upon treatment with Notch inhibitors DAPT and RO4929097. (B) DU145 cells migration was similarly scored in cells stably expressing shPlexinD1, shNotch1 or shScr. Mean ± SD is shown. (C-D) PlexinD1 expression in PC3 cells was knocked-down by stable expression of two independent shRNA constructs, indicated as #48 and #52 (C; see Methods), and the migration of these cells was assessed by Boyden chamber assay (D). (E-F) Boyden chamber migration assays with PC3 cells subjected to PlexinD1 knock-down by siRNAs (directed against 3’ untranslated sequence) and subsequently transfected with non-targetable PlexinD1 cDNA construct to achieve re-expression (and relative control conditions); representative images (E) and quantitative analysis (F). Mean ± SD is shown.
(PDF)

S7 Fig Correlation of Slug expression with PlexinD1 and Notch1 in human prostate cancer. (A) Correlation analysis of mRNA levels of Slug (*SNAI2* gene symbol) and either Notch1 or PlexinD1 in TCGA prostate cancer dataset (n = 499). Spearman coefficient and p values are indicated in the graph. (B) Correlation of Slug and PlexinD1 mRNA levels in GEO—GSE54460 prostate cancer dataset (n = 106). Differential gene expression is indicated as Log2 values on either axis.
(PDF)

S8 Fig Regulation of E cadherin levels by PlexinD1 signaling. (A) E-cadherin expression levels were analyzed by immunoblotting in PC3 cells subjected to PlexinD1 knock-down by siRNAs and/or subsequently transfected with non-targetable PlexinD1 cDNA construct to achieve re-expression (same conditions as analyzed in Fig 6E and 6F). (B) Functional rescue experiment similar to that in A, by re-expressing wild-type or RA-mutated PlexinD1 constructs in gene silenced cells (by siRNAs); PlexinD1 and E cadherin levels were revealed by immunoblotting.
(PDF)

S9 Fig Regulation of E cadherin levels by PlexinD1 in vitro and in tumor xenografts in vivo. (A) Q-PCR analysis of E cadherin mRNA levels in PC3 cells stably expressing shPlexinD1. (B) IHC analysis of E cadherin expression in tumor xenografts formed by the same cells analyzed in A.
(PDF)
