# Correction: PlexinD1 Is a Novel Transcriptional Target and Effector of Notch Signaling in Cancer Cells

**DOI:** 10.1371/journal.pone.0168429

**Published:** 2016-12-09

**Authors:** The PLOS ONE Staff

The following information is missing from the Funding section: This study was supported by The Italian Ministry of Health (Ricerca Corrente 2016). The publisher apologizes for the error.
