# Supplementary figures and images for: PlexinD1 Is a Novel Transcriptional Target and Effector of Notch Signaling in Cancer Cells
Source: PLoS One. 2016 Oct 17;11(10):e0164660. doi: 10.1371/journal.pone.0164660 (PMC5066946; doi:10.1371/journal.pone.0164660)

Supplementary Figure S1

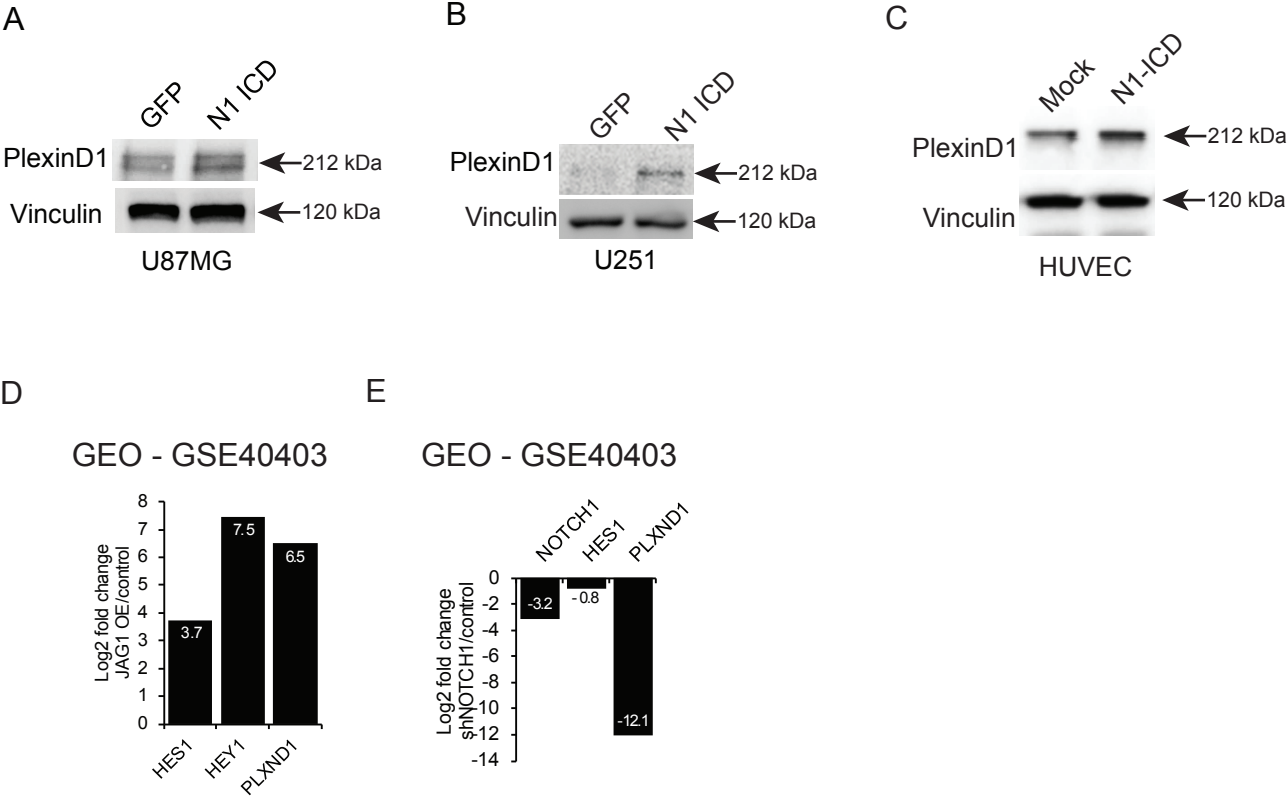

Supplement: S1 Fig — (A, B) Constitutively active Notch1-intracellular domain (N1-ICD) was overexpressed in glioblastoma-derived cell lines U87 and U251, and PlexinD1 protein expression was determined in cell lysates by immunoblotting. Vinculin levels were analyzed to provide reference for protein loading. (C) PlexinD1 protein detection by immunoblotting in HUVEC cells overexpressing N1-ICD. (D) GEO dataset GSE40403 was analyzed for expression of HES1, HEY1 and PLXND1 in HUVEC cells overexpressing Notch ligand Jag1 (D) or subjected to NOTCH1 knock-down (E). (PDF) [file pone.0164660.s001.pdf]

Supplementary Figure S2.

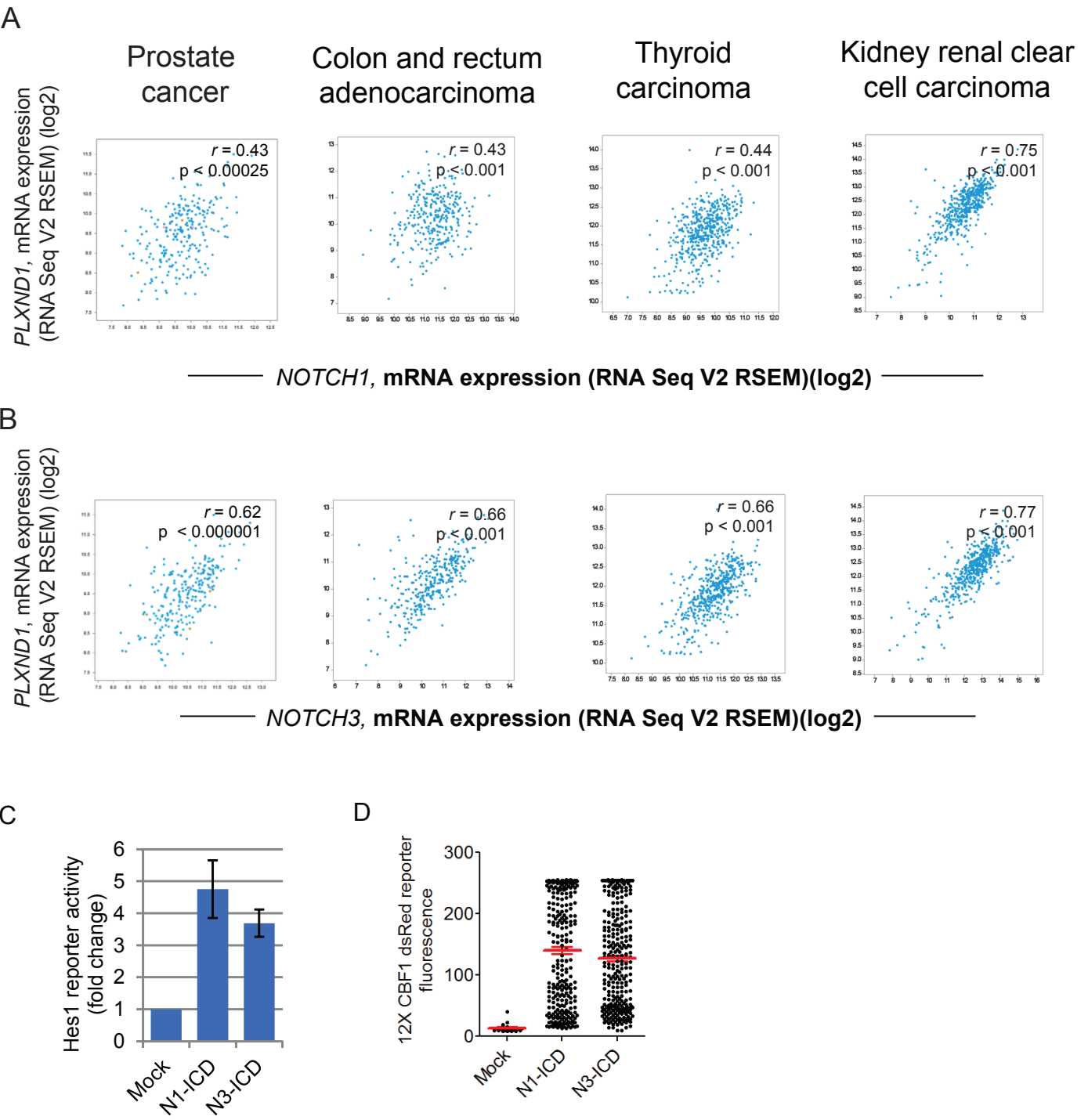

Supplement: S2 Fig — (A-B) TCGA datasets of prostate, colon and rectum adenocarcinoma, thyroid and kidney renal cell carcinoma were analyzed using cBioportal interface for Notch1 (or Notch3, respectively) and PlexinD1 expression levels; two-gene correlations were plotted, also indicating Spearman correlation coefficients (r). p value was calculated from the panel of ‘mutual exclusivity and co-occurrence analysis’. (C) 293T cells were transiently transfected with mock plasmid, N1-ICD or N3-ICD in combination with Hes1 Luc reporter plasmid. 48 hrs after transfection, cells were lysed and Hes1-luc reporter activity was measured. Mean values ± SD are shown. (D) COS7 cells were transfected with 12X CBF dsRed reporter in combination with mock plasmid, N1-ICD and N3-ICD. Mean ± SD is shown. (PDF) [file pone.0164660.s002.pdf]

Supplementary Figure S3.

A

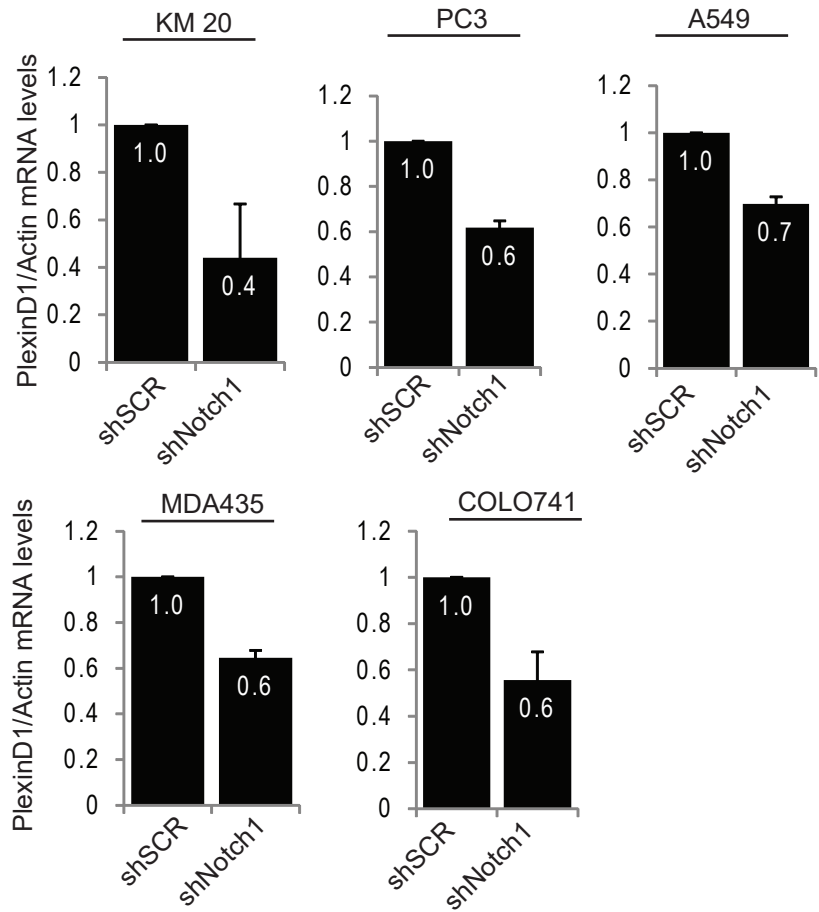

B

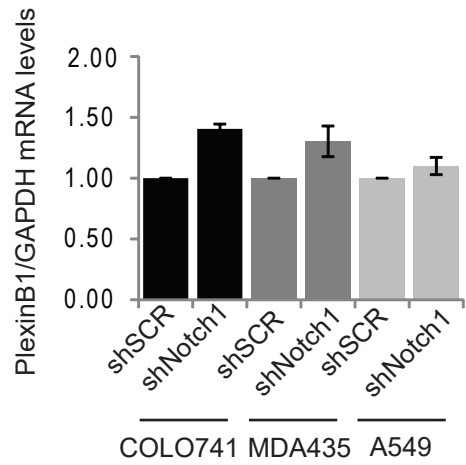

C

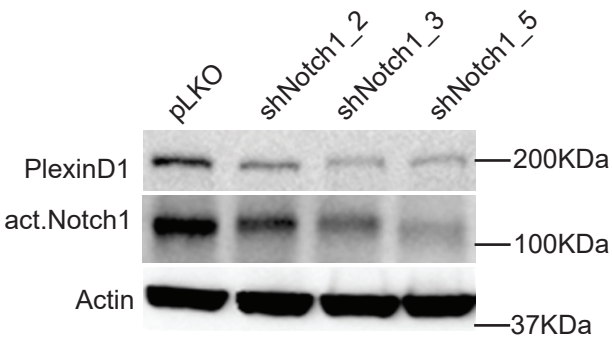

Supplement: S3 Fig — (A) PlexinD1 mRNA levels were analyzed in KM20, PC3, A549, COLO741, MDA435 cancer cells stably expressing shNotch1 or shScr. Relative gene expression was normalized to control cells. (B) PlexinB1 mRNA levels were analyzed by qPCR in the indicated tumor cells expressing shNotch1 (or shScr). (C) Three independent shRNAs targeting Notch1 were transfected in PC3 cells to validate the specific effect of this knock down on PlexinD1 mRNA levels. Bar graphs show mean values ± SD. (PDF) [file pone.0164660.s003.pdf]

Supplementary Figure S4.

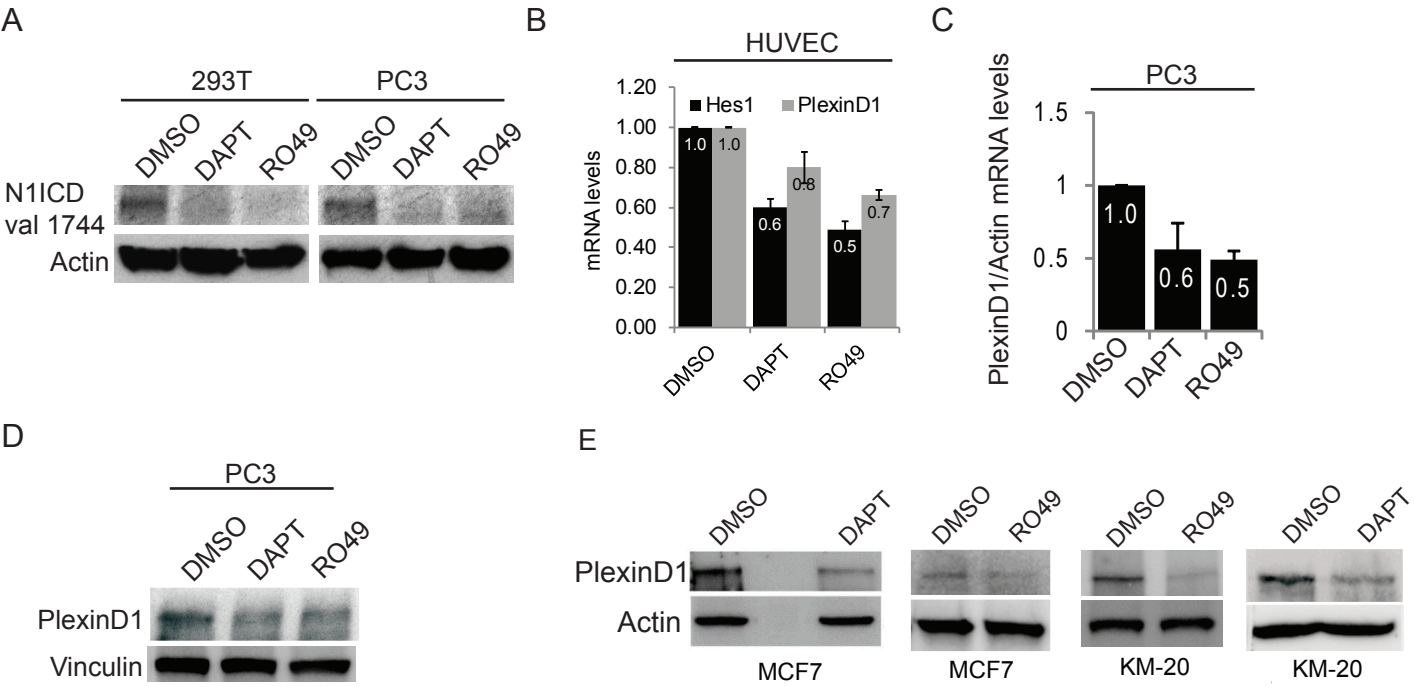

Supplement: S4 Fig — (A) The presence of activated Notch1 intracellular cleaved domain (N1-ICD) in 293T and PC3 cells was revealed by immunoblotting with an isoform specific anti-Val1744 antibody; N1-ICD levels dramatically dropped in cells treated with (γ-secretase) Notch cleavage inhibitors DAPT (25μM) or RO4929097 (25μM). (B) The mRNA levels of Notch target genes HES1 and PLXND1 were analyzed by qPCR in HUVEC endothelial cells, in basal conditions and upon treatment with Notch inhibitors DAPT or RO4929097. (C-D) PC3 cells were treated with DAPT (25μM) and RO4929097 (25μM) for 72 hrs and PLXND1 mRNA were analyzed by qPCR (C); independently, protein lysates were analyzed for PlexinD1 and vinculin by immunoblotting (D). (E) MCF7 and KM20 carcinoma cells were treated with Notch inhibitors DAPT or RO4929097 for 72 hrs (in independent experiments), and cell lysates were analyzed by immunoblotting to reveal PlexinD1 expression levels. (PDF) [file pone.0164660.s004.pdf]

Supplementary Figure S5.

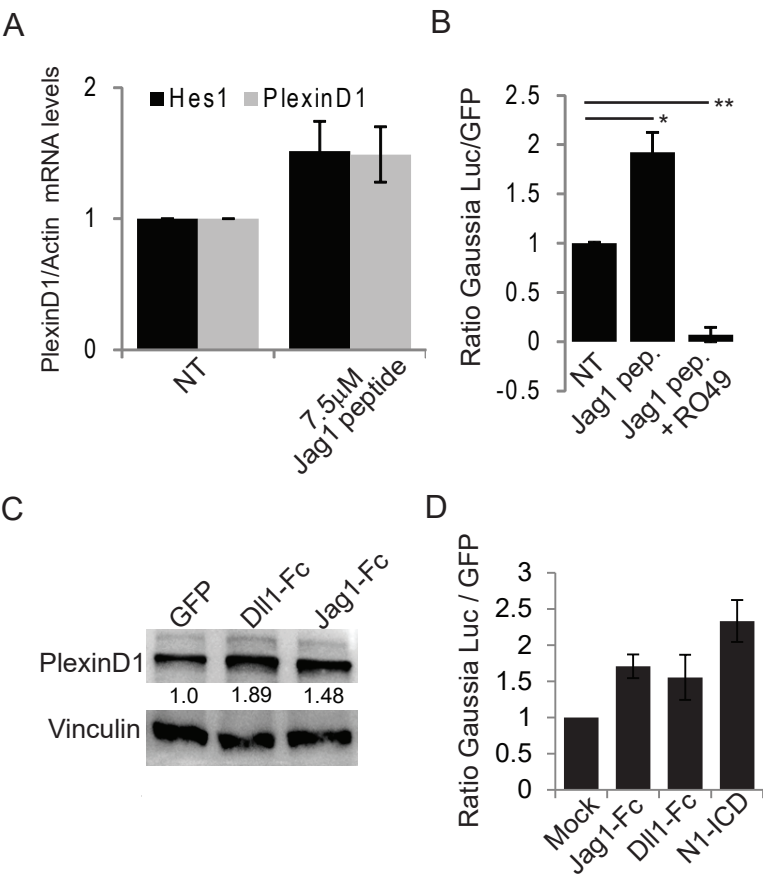

Supplement: S5 Fig — (A) PC3 cells were treated with 7.5 μM Jag1 soluble peptide for 24hrs and compared with untreated control cells. PlexinD1 and Hes1 mRNA levels were analyzed by qPCR. (B) PC3 cells were transfected with PlexinD1 promoter reporter construct (as in main Fig 2); the following day the cells were treated with Jag1 peptide 7.5 μM or Jag1 peptide plus Notch inhibitor RO4929097 (25μM), and after 24hrs cell-conditioned media were analyzed to reveal Gaussia luciferase activity. (C) PC3 cells were transiently transfected with either GFP, Dll1-Fc or Jag1-Fc; 48 hours later, PlexinD1 and vinculin levels were analyzed by immunoblotting; relative band intensity was quantified and normalized to controls. (D) PC3 cells were transfected with PlexinD1 promoter reporter construct in combination with Dll1-Fc, Jag1-Fc and N1-ICD. Mean ± SD is shown. (PDF) [file pone.0164660.s005.pdf]

Supplementary Figure S6

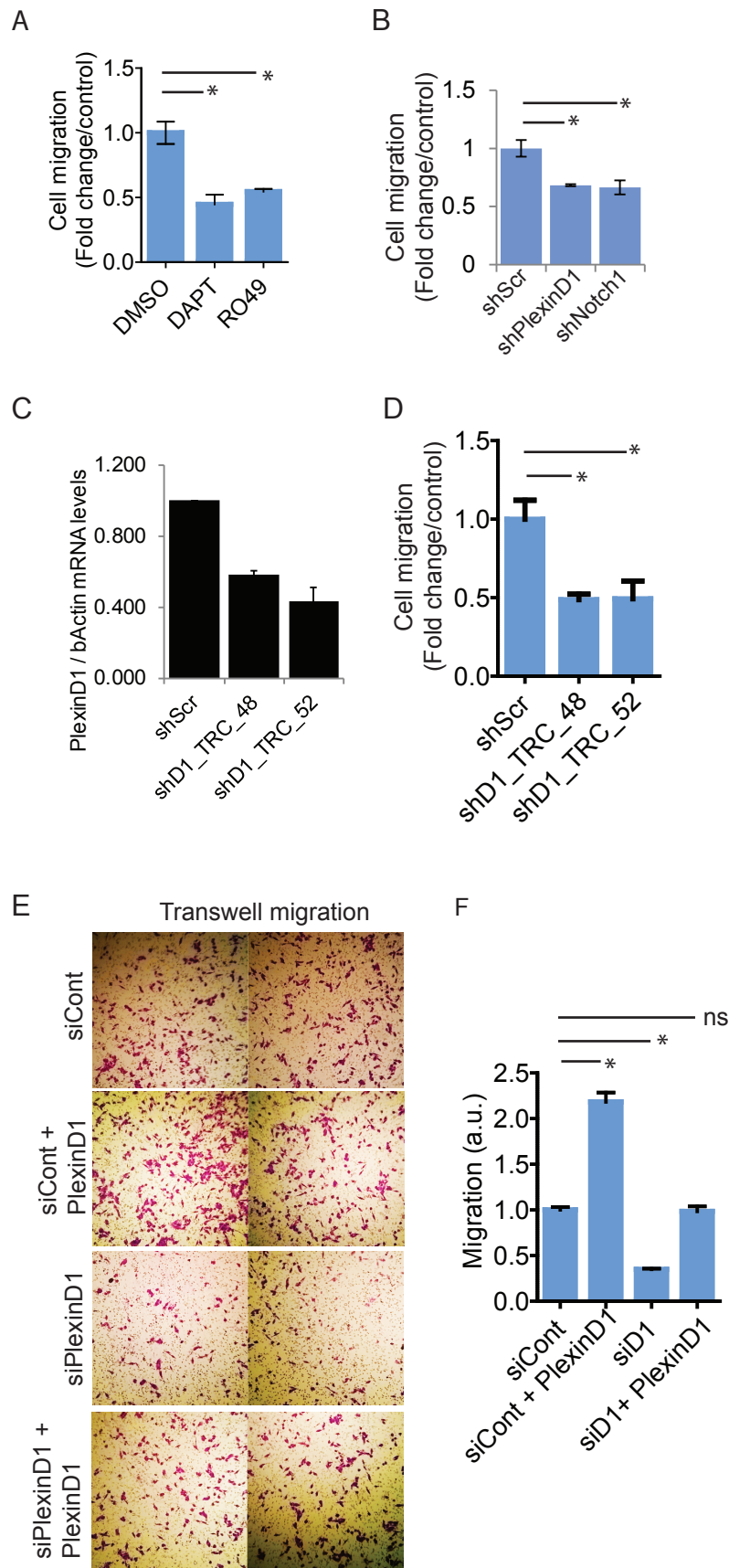

Supplement: S6 Fig — (A) Analysis of DU145 prostate cancer cells migration (in transwell Boyden Chamber assays) upon treatment with Notch inhibitors DAPT and RO4929097. (B) DU145 cells migration was similarly scored in cells stably expressing shPlexinD1, shNotch1 or shScr. Mean ± SD is shown. (C-D) PlexinD1 expression in PC3 cells was knocked-down by stable expression of two independent shRNA constructs, indicated as #48 and #52 (C; see Methods), and the migration of these cells was assessed by Boyden chamber assay (D). (E-F) Boyden chamber migration assays with PC3 cells subjected to PlexinD1 knock-down by siRNAs (directed against 3’ untranslated sequence) and subsequently transfected with non-targetable PlexinD1 cDNA construct to achieve re-expression (and relative control conditions); representative images (E) and quantitative analysis (F). Mean ± SD is shown. (PDF) [file pone.0164660.s006.pdf]

Supplementary Figure S7

A

TCGA - prostate cancer provisional (n = 499)

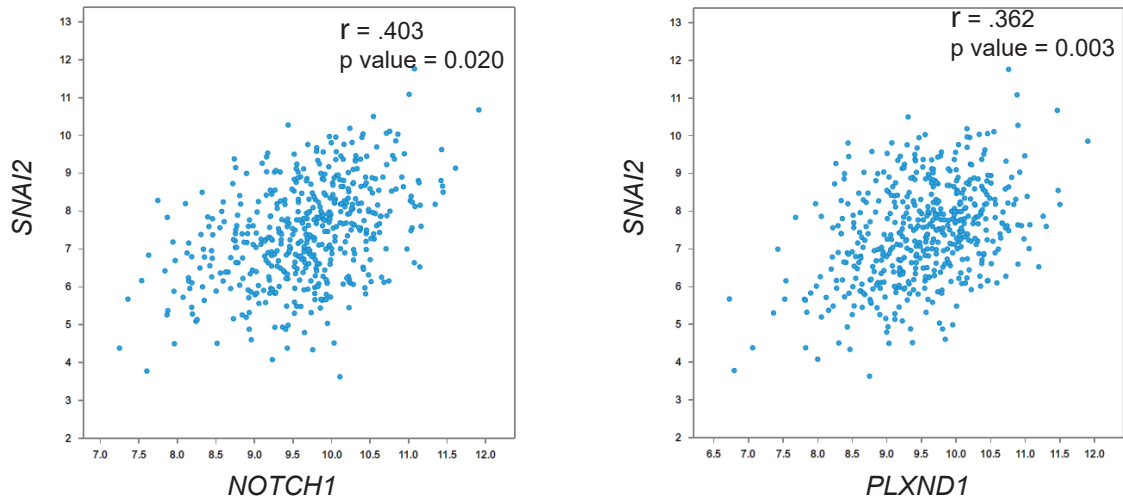

B

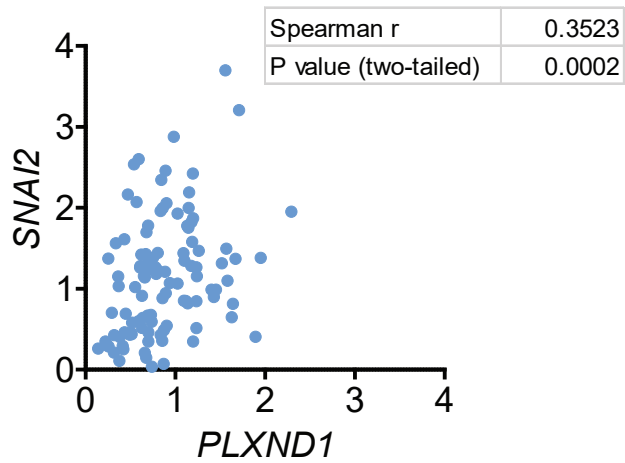

Supplement: S7 Fig — (A) Correlation analysis of mRNA levels of Slug (SNAI2 gene symbol) and either Notch1 or PlexinD1 in TCGA prostate cancer dataset (n = 499). Spearman coefficient and p values are indicated in the graph. (B) Correlation of Slug and PlexinD1 mRNA levels in GEO—GSE54460 prostate cancer dataset (n = 106). Differential gene expression is indicated as Log2 values on either axis. (PDF) [file pone.0164660.s007.pdf]

Supplementary Figure S8

A

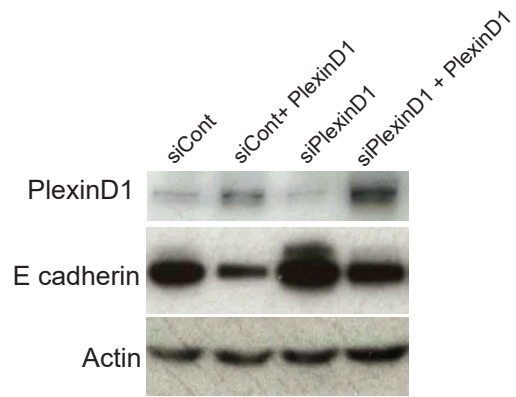

B

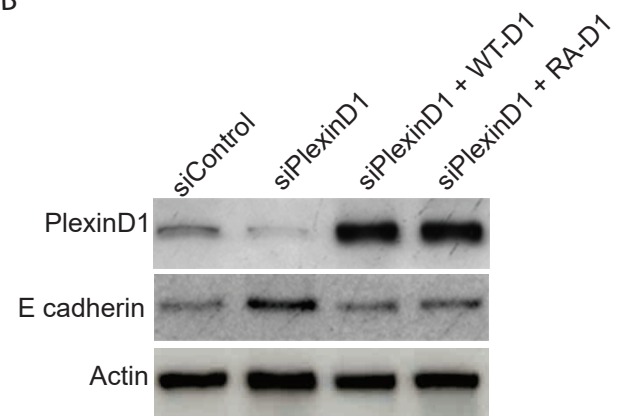

Supplement: S8 Fig — (A) E-cadherin expression levels were analyzed by immunoblotting in PC3 cells subjected to PlexinD1 knock-down by siRNAs and/or subsequently transfected with non-targetable PlexinD1 cDNA construct to achieve re-expression (same conditions as analyzed in Fig 6E and 6F). (B) Functional rescue experiment similar to that in A, by re-expressing wild-type or RA-mutated PlexinD1 constructs in gene silenced cells (by siRNAs); PlexinD1 and E cadherin levels were revealed by immunoblotting. (PDF) [file pone.0164660.s008.pdf]

Supplementary figure S9

A

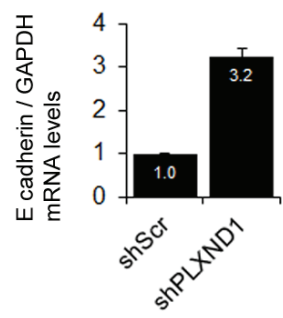

B

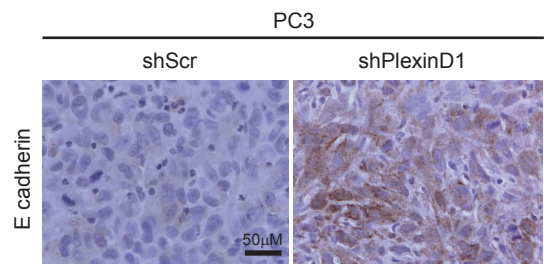

Supplement: S9 Fig — (A) Q-PCR analysis of E cadherin mRNA levels in PC3 cells stably expressing shPlexinD1. (B) IHC analysis of E cadherin expression in tumor xenografts formed by the same cells analyzed in A. (PDF) [file pone.0164660.s009.pdf]
